# Effects of foam rolling on hamstrings stiffness in damaged and non-damaged muscle states

**DOI:** 10.3389/fphys.2024.1396361

**Published:** 2024-04-08

**Authors:** Rok Vatovec, Anja Grandovec, Žiga Kozinc, Matej Voglar

**Affiliations:** University of Primorska, Faculty of Health Sciences, Izola, Slovenia

**Keywords:** exercise-induced muscle damage, muscle stiffness, recovery strategies, myofascial release, delayed-onset of muscle soreness

## Abstract

**Introduction:** The aim of this study was to examine the effects of foam rolling (FR) on hamstring muscles stiffness in both non-damaged and exercise-induced muscle damage (EIMD) states, using shear wave ultrasound elastography to measure changes in shear modulus.

**Methods:** Fourteen healthy adults (25.5 ± 4.7 years) participated in a within-participant repeated measures design, with a 2-minute FR intervention applied on one leg and contralateral leg serving as a control. The damaging protocol encompassed maximal eccentric knee extensions performed on an isokinetic dynamometer and the Nordic hamstring exercise, consisting of 3 sets of 10 and 6 repetitions, respectively. Measurement were taken at baseline and then 1 h, 24 h and 48 h after the damaging protocol.

**Results:** The results indicated no significant time × leg interaction for shear modulus in biceps femoris, semimembranosus, and semitendinosus muscles in both non-damaged and damaged states. Notably, there was a significant increase in biceps femoris (*p* = 0.001; η^2^ = 0.36) and semitendinosus (*p* < 0.001; η^2^ = 0.44) shear modulus after EIMD, but no significant differences were found between the FR and control leg, which was also the case for muscle soreness, range of motion, and passive resistive torque (*p* = 0.239–0.999 for interactions).

**Discussion:** The absence of significant changes post-FR intervention suggests a limited role of short-duration FR in altering muscle stiffness during recovery from EIMD. These findings contribute to the understanding of FR’s role in muscle recovery. Although this was not directly investigated, our results suggest a predominance of central mechanisms rather than direct mechanical modifications in muscle properties. This research highlights the necessity for additional investigations to explore how FR interventions influence muscles in different states and to elucidate the mechanisms underlying these influences.

## 1 Introduction

Exercise-induced muscle damage (EIMD) and associated delayed onset muscle soreness (DOMS) significantly impact athletes (Clarkson and Hubal, 2002; Cheung et al., 2003), particularly following eccentric exercise. In addition to causing pain, EIMD also impairs physical performance (Mancinelli et al., 2006), which underscores the need for effective recovery strategies. Several recovery strategies, such as stretching (Afonso et al., 2021), whole-body cryotherapy (Hausswirth et al., 2011), the use of compression garments (Brown et al., 2017), and targeted nutritional and supplementation strategies (Harty et al., 2019) have been investigated. Recently, foam rolling (FR) has gained prominence in sports settings for its ability to enhance range of motion (RoM) and reduce muscle stiffness without detrimentally affecting muscle strength and athletic performance (Wiewelhove et al., 2019; Behm et al., 2020).

Recent research has explored the efficacy of FR for speeding up recovery after EIMD. Nakamura et al. (2021a) demonstrated that a brief, 90-s session of FR significantly reduced muscle soreness and improved muscle strength when applied 48 h post-exercise. Similarly, Naderi et al. (2020) observed that FR not only decreased muscle pain but also enhanced joint proprioception and reduced force loss in sport science students after eccentric exercise. D’Amico et al. (2020) noted that while FR may reduce soreness following damaging exercise, its effects do not seem to be mediated by the autonomic nervous system. Romero-Moraleda et al. (2019) compared vibration FR and non-vibration FR, finding that the former offered greater short-term benefits in pain perception and hip joint RoM, although both methods were effective in improving overall muscle recovery. Additionally, numerous studies have indicated that FR can contribute to a faster recovery in vertical jump height and performance in other explosive activities (Macdonald et al., 2014; Pearcey et al., 2015; Rey et al., 2019; Romero-Moraleda et al., 2019; D’Amico et al., 2020), which may be particularly important during competitions periods with demanding schedules. Research also suggests that a short duration of FR, approximately 90 s, may be enough to produce these beneficial effects (Hughes and Ramer, 2019). This is a crucial factor for athletes who often have limited time for recovery in athletic environments.

While the literature on the effect of FR on recovery after EIMD is abundant, the mechanisms underlying these benefits are not fully understood (Drinkwater et al., 2019). Both peripheral (e.g., increased blood flow; alteration of musculotendinous tissue properties) and central (pain modulation) mechanisms have been proposed (Macdonald et al., 2014; Pearcey et al., 2015; Behm and Wilke, 2019). The role of the central mechanisms is supported by evidence indicating that FR can affect the contralateral limb, suggesting systemic (rather than purely local) effects (Aboodarda et al., 2015). However, despite these insights, a comprehensive understanding of the precise mechanisms by which FR facilitates recovery from EIMD is still developing. Recently, shear wave ultrasound elastography has emerged as a valuable tool for assessing muscle stiffness and understanding the mechanical responses to eccentric exercise (Ličen and Kozinc, 2022). Several studies have demonstrated increased shear modulus (an index of stiffness) after damaging exercise (Lacourpaille et al., 2014; 2017; Agten et al., 2017; Goreau et al., 2022). On a non-damaged hamstring muscles, a study by Morales-Artacho et al. (2017) investigated the effects of FR on shear modulus and reported short-term reductions in muscle stiffness, a finding later confirmed for quadriceps muscle as well (Reiner et al., 2021). However, these studies did not explore how FR affects stiffness in muscles recovering from EIMD. This leaves a significant gap in the understanding of the role of FR in muscle recovery, particularly in the context of muscle stiffness changes during the recovery process. One study investigated the effects of FR on rectus femoris stiffness after resistance training, reporting no difference between legs that did or did not receive the treatment (Schroeder et al., 2019). However, the extent of EIMD in this study was not clearly established. Furthermore, the authors employed myotonometry for assessing muscle stiffness, a technique that has demonstrated limited agreement with ultrasound elastography in evaluating thigh muscles (Bravo-Sánchez et al., 2021; Lee et al., 2021).

The aim of this study was to address the knowledge gap on the impact of a FR bout on muscle stiffness during recovery, particularly in muscles exhibiting EIMD. Based on the previous literature showing potential benefits of relatively short FR sessions (Hughes and Ramer, 2019), a 2-min FR intervention was investigated in this study. The primary outcome of interest was the change in shear modulus in both non-damaged muscles and muscles post-EIMD. The null hypothesis was that FR does not lead to a significant change in shear modulus in both non-damaged and post-EIMD muscles during the recovery phase, while the alternative hypothesis asserts that FR significantly reduces the shear modulus in these muscle states before and during recovery. Additionally, this study included secondary outcomes, including the passive resistive torque (PRT), RoM, and perceived soreness in the muscles, to obtain a more comprehensive understanding of the effects of FR. The findings of this study are anticipated to provide valuable insights into the mechanisms underpinning the role of FR in muscle recovery, expanding upon existing literature on the effects of FR on muscle stiffness in non-damaged muscles (Morales-Artacho et al., 2017; Nakamura et al., 2021b).

## 2 Methods

### 2.1 Participants

We used G*Power 3.1 software (Heinrich Heine University, Düsseldorf, Germany) for calculating required sample size for the study. Drawing on prior research, large changes in shear modulus due to eccentric exercise were anticipated (Ličen and Kozinc, 2022). Furthermore, moderate to substantial effects of FR on shear modulus were also expected (Morales-Artacho et al., 2017). The total sample size necessary to confirm our alternative hypothesis was therefore calculated based on an anticipated medium effect size (Cohen’s f = 0.4), with an alpha level set at 0.05 and a statistical power of 80%. This calculation was specifically done for a general linear model for repeated measures with two levels, focusing on interaction effects. The sample size calculation indicated that >11 participants were needed for the study. The study involved fourteen healthy individuals (7 men, age = 26.5 ± 4.1 years, body mass = 79.3 ± 12.8 kg, height = 181.9 ± 9.5 cm; 7 women, age = 24.5 ± 3.8 years, body mass = 61.2 ± 11.0 kg, height = 166.1. ± 7.8 cm), comprising an equal number of males and females. Eligibility for participation required regular physical activity engagement (minimum of 3 hours per week) and age between 18 and 40 years. Exclusion criteria included recent musculoskeletal injuries or pain in the lower extremity or trunk (within the last 6 months), active participation in competitive sports, or the presence of significant cardiovascular or systemic illnesses. Participants provided written consent after being briefed about the objectives and potential risks associated with the study. The research adhered to the principles of the Helsinki Declaration and received approval from the National Medical Ethics Committee (approval number: 0120-557/2017/4).

### 2.2 Study design

The study was designed as a within-participant repeated measures experiment conducted over three sessions. In this design, one leg of each participant received the FR intervention while the other leg served as a control. The legs were randomly allocated to intervention and control conditions for each participant. In the first session, participants underwent baseline measurements, including shear wave elastography, RoM, and PRT assessments. Prior to baseline measurements, the participants laid on the therapeutic table in the position used for shear modulus assessments (described later) for approximately 15 min. During this period the researchers determined the location and orientation of the ultrasound probe. It has to be admitted that this study design presents a potential bias due to contralateral effects, a phenomenon documented in previous research primarily attributed to central or psychological factors, such as pain tolerance. However, considering our focus on muscle stiffness—a peripheral measure—the utilization of the contralateral leg as a control does not present a methodological concern. In other words, the direct measurement of physical properties such as muscle stiffness should remain unaffected due to their localized nature.

**FIGURE 1:**
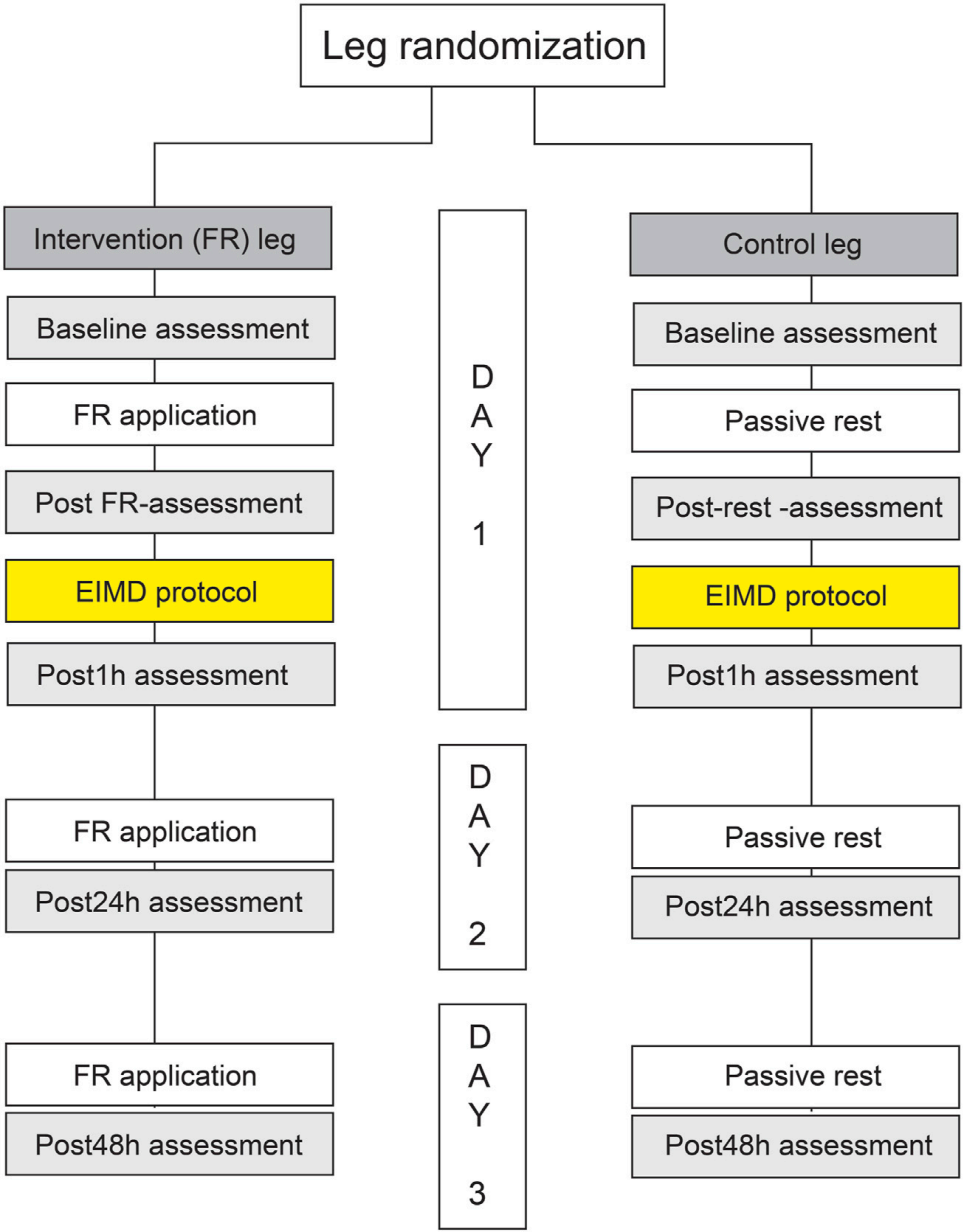
The flowchart of the study.

Baseline measurements were followed by a warm-up (10 min of stationary cycling at an intensity of 1.5 W/kg and a cadence of 90 bpm) and a bout of FR, and another round of measurements (postFR). These data were crucial for assessing the acute effects of FR on a non-damaged muscle. After these second measurements, the participants engaged in an eccentric exercise protocol and another set of measurement was performed 1 h after (Post1h). The second session, scheduled precisely 24 h after the first, involved another FR bout, followed by subsequent measurements (Post 24 h). This procedure was repeated 48 h after the first session (Post 48 h). The study flowchart is shown in Figure 1.

### 2.3 Measurement procedures

#### 2.3.1 Shear-wave elastography

The shear modulus of the hamstring muscles (biceps femoris - BF, semitendinosus - ST, and semimembranosus - SM) was assessed using shear-wave elastography via an ultrasound system (Resona 7, Mindray, Shenzen, China). We employed the sound touch quantification mode for its capability to directly quantify shear modulus values. The ultrasound was configured to the musculoskeletal SWE mode, which is based on a tissue density assumption of 1000 kgm^-3^. A medium-sized linear probe (Model L11-3U, Mindray, Shenzhen, China) was used, with a substantial amount of water-soluble, hypoallergenic ultrasound gel (AquaUltra Basic, Ultragel, Budapest, Hungary). The target area was defined as a 1 × 1 cm region of interest. We adjusted the depth for each participant to ensure exclusive capture of muscle tissue, and this depth was maintained consistently across sessions.

**FIGURE 2:**
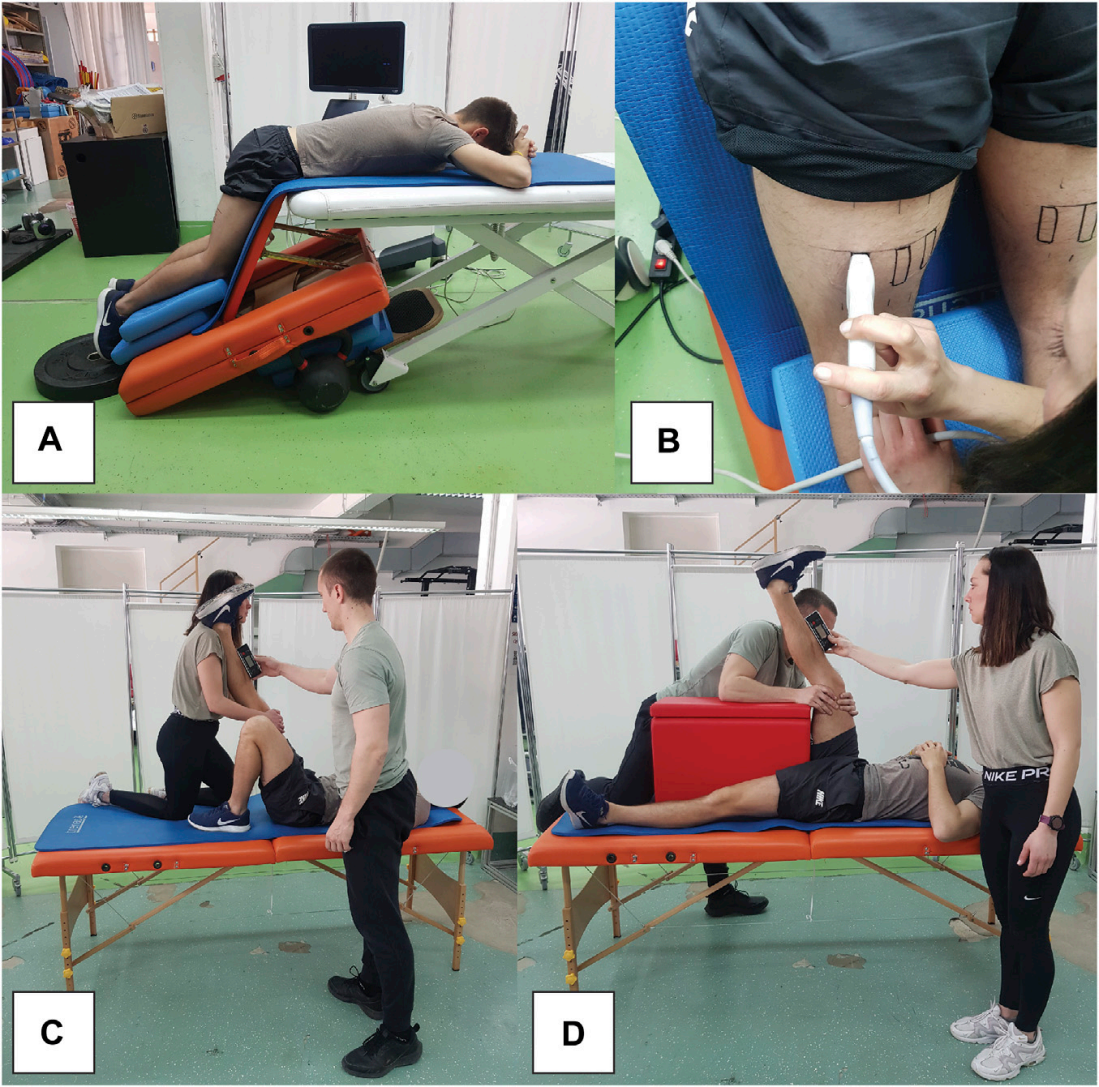
Snapshots of measurement procedures, showing participant position during elastography assessments **(A)**, elastography probe positioning **(B)**, as well as passive **(C)** and active **(D)** range of motion assessments.

Participants were placed in a prone position on the edge of a therapy table, with their hips and knees flexed at 60° and 30°, respectively (Figure 2A). This was done to increase the sensitivity of the measurements, as previous studies indicate larger and more consistent shear modulus increases when assessed at longer muscle lengths (Lacourpaille et al., 2014; 2017; Xu et al., 2019). To account for potential stretch-relaxation effects, participants remained in this position for 5 minutes before each measurement. Additionally, we maintained a constant room temperature of 20°C to control for its potential impact on muscle stiffness. We marked the exact location and orientation of the probe on the skin with a permanent marker before baseline measurements. The probe was positioned midway between the ischial tuberosity and the popliteal fossa (Figure 2B). The muscles were first viewed in B-mode in the transverse plane to help determine the exact width of each muscle. Afterwards, the probe was oriented longitudinally to follow the line of the muscle fascicles and rotated to ensure uninterrupted visualization of the fascicles and superficial fascia. For each measurement, eight consecutive scans were conducted, and the median of two measurements at each time point was used for analysis. This approach, contrasting with previous studies, incorporates the median value due to its reduced sensitivity to potential outliers in measurements.

#### 2.3.2 Passive resistive torque

Our preliminary data analysis (Voglar et al., 2022), alongside previous studies (Chino and Takahashi, 2018) suggests a lack of correlation between the responses in shear modulus and PRT after eccentric exercise. Consequently, we incorporated PRT measurements to more comprehensively capture the spectrum of changes post-exercise and the impact of FR. Namely, elastography enables direct measurements of muscles shear modulus in selected regions of interest, while PRT measurements offer a holistic perspective, integrating the resistance of the entire joint complex (including the tendons, ligament and other tissues).

For the PRT evaluation, the participants were seated on an isokinetic dynamometer (Humac Norm, Computer Sports Medicine Inc., Massachusetts, US), with their hips flexed at 90°. The upper body was secured using the device’s harness, and straps were applied to anchor the pelvis and the upper part of the thigh, just proximal to the knee joint. The dynamometer’s rotation axis was aligned with the knee’s lateral epicondyle, and the lever arm was attached 2 cm proximal to the malleoli using a strap. Before starting the measurements, the range of movement was determined, and a correction was applied within the software to account for the weight of the limb being tested. To minimize reflexive or voluntary muscle contractions, the measurements were conducted at a slow angular velocity of 5°/s, and participants were instructed to remain relaxed during the process. The passive isokinetic torque was recorded within a knee flexion range of 70°–0°. Each participant underwent a preliminary set of three cycles to become accustomed to the procedure. The actual measurement consisted of five consecutive cycles of passive movement, with the middle three cycles being selected for detailed analysis. Peak passive knee flexor torque was calculated as the average passive torque in the last 5° of terminal extension (0°–5°). (Seymore et al., 2017).

#### 2.3.3 Range of motion

Passive and active knee flexion RoM (Figures 2C, D, respectively) were assessed using the passive straight leg raise test and the active knee extension test, respectively. A digital inclinometer (Baseline Digital Inclinometer, Fabrication Enterprises, White Plains, United States) was positioned at the midpoint of the tibia, equidistant from the medial joint line of the knee and the medial malleolus. During the passive RoM assessment, participants were positioned supine on a therapy table, instructed to remain fully relaxed. One researcher ensured the knee being tested was completely extended and then proceeded to flex the hip passively, while maintaining the opposite thigh stabilized. The moment the pelvis began to tilt posteriorly excessively, as identified by palpation in the lumbosacral area, was marked as the ROM’s endpoint. In the active RoM test, participants were placed supine on an exercise mat. The researcher fixed the hip of the leg under examination at a 90-degree angle, while the other leg remained stable on the table. Participants were then asked to actively straighten their knee to the maximum extent possible. The RoM was recorded using the inclinometer by the second researcher. Each leg underwent two trials, with the mean of these two trials being used for subsequent analysis.

#### 2.3.4 Pain

Pain due to DOMS was assessed with visual analogue scale. To ensure consistency in the scale’s interpretation, participants were informed that the scale ranged from 0, indicating no sensation, to 10, representing the most intense sensation conceivable (Romero-Moraleda et al., 2019). Participants reported their pain level prior to the second and third session. The participants were not informed on their reported pain level from the previous session. Pain was evaluated only in the passive resting condition.

### 2.4 Eccentric exercise protocol

**FIGURE 3:**
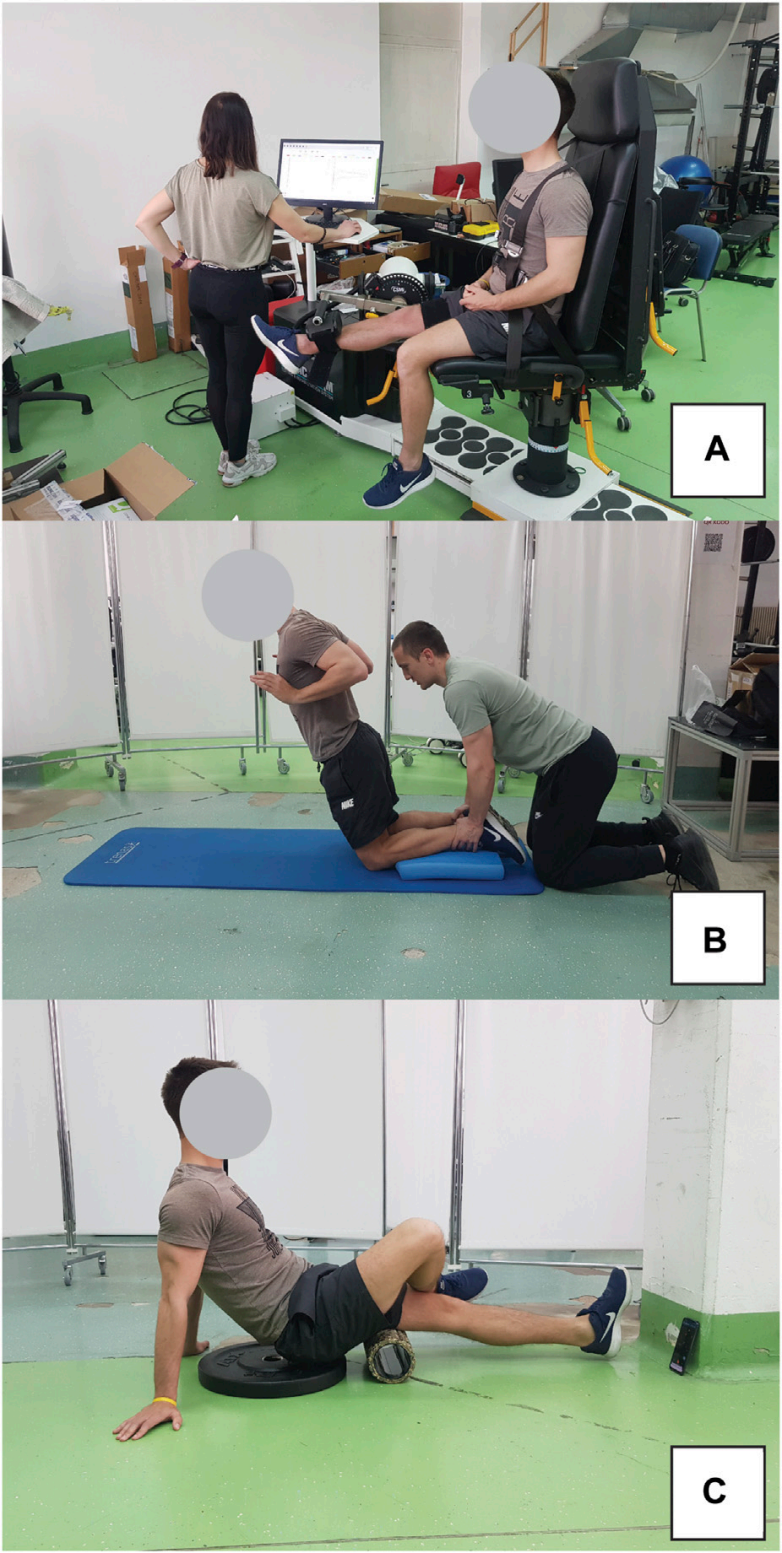
Exercise protocol for inducing muscle damage included eccentric isokinetic contractions on dynamometer **(A)** and Nordic hamstring exercise **(B)**. Participant position during foam rolling intervention is also shown **(C)**.

Eccentric exercise protocol included maximal eccentric knee extensions using an isokinetic dynamometer (Humac Norm, Computer Sports Medicine Inc., Massachusetts, US) in seated position (hips in 90° of flexion), as well as the Nordic hamstring exercise. The isokinetic exercise involved 3 sets of 10 repetitions, with 2-min rest intervals. During the eccentric phase, participants were instructed to maximally resist the dynamometer by pulling their heel towards their buttock. In contrast, the concentric phase required minimal effort in the same movement (Figure 3A). Subsequent to this protocol, the participants executed 3 sets of 6 repetitions of the Nordic hamstring exercise (Figure 3B), with 2 minutes of rest between each set. They knelt on a foam pad, while a researcher stabilized their shins using his body weight. The exercise entailed a slow, controlled descent of the body towards the ground. Participants were allowed to perform the exercise with their hips flexed up to 45°, allowing for controlled movement over an extended RoM.

### 2.5 Foam rolling intervention

The FR intervention was conducted using a hard foam roller featuring a smooth texture, following the recommendations from the literature (Behm et al., 2020). Two 1-min bouts were performed, separated by a 1-min rest interval. The pacing of the activity was regulated using a smartphone metronome app, set to 27 beats per minute (one beat to complete one direction of movement). Subjects were advised to apply maximal pressure on the leg receiving the treatment during FR. To enhance this pressure, the non-treated leg was crossed over and positioned on top of the treated leg. The application of FR extended from the ischial tuberosity down to the popliteal fossa. The participant positon during FR intervention is shown in Figure 3C.

### 2.6 Statistical analysis

We analysed the collected data using the IBM SPSS (version 25.0) software. Descriptive statistics are presented as the mean and standard deviation. The normality of the data distribution was checked using the Shapiro-Wilk test and a visual assessment of histograms. Reliability between repetitions was assessed with intraclass correlation coefficient (ICC; two-way mixed, absolute agreement) and coefficient of variation (CV). ICC values <0.5 were considered indicative of poor reliability, values between 0.5 and 0.75 as moderate reliability, values between 0.75 and 0.9 as good reliability, and values above 0.90 as excellent reliability (Koo and Li, 2016). CVs were and interpreted as poor (CV >10%), moderate (CV = 5–10%), or good (CV <5%) (Banyard et al., 2017). To assess the effects of FR on a non-damaged muscle, a general linear model for repeated measures with time (baseline, post-FR) and leg (intervention, control) was applied. Similarly, to assess the effects of FR on damaged muscles, a general linear model for repeated measures with time (baseline, post-1h, post-24 h and post48 h) and leg (intervention, control) was used. The primary interest was in the interaction effects, in order to examine whether the change over time differs between the two legs. This is crucial for establishing whether FR has a distinct effect on muscle recovery compared to the control condition. The effect sizes were expressed as partial η^2^ and interpreted as trivial (<0.02), small (0.02–0.13), moderate (0.14–0.26) and large (>0.26) (Bakeman, 2005). Post-hoc tests with Bonferroni correction were applied in case of statistically significant main effects. The associations between changes in shear modulus, PRT and RoM were examined with Pearson’s correlation coefficient r). The correlations were interpreted as negligible (r < 0.1), weak (r = 0.1–0.4), moderate (r = 0.4–0.7), strong (r = 0.7–0.9) and very strong (r = 0.9) (Akoglu, 2018). Statistically significant differences were accepted at a confidence level of α < 0.025 (accounting for two separate analysis to control for type 1 error risk).

## 3 Results

### 3.1 Reliability

Reliability analysis is available in Supplementary Material S1. Overall, ICC values ranged widely indicating a varied level of agreement between repetitions for different variables across legs and different time points. Most variables demonstrated high reliability with ICCs greater than 0.80, particularly notable in the post-intervention measures where ICC values often exceeded 0.90, signifying excellent reliability. Conversely, certain conditions exhibited lower reliability. Only one variable (BF shear modulus at post-24 h on the intervention leg) exhibited poor reliability) as evidenced by ICC at 0.40. CV values ranged from 1.11% to 14.94%, reflecting good to low variability across outcome variables.

### 3.2 Effects of FR in non-damaged muscle state

Analysis of the effect of FR on non-damaged muscles is shown in Table 1. Analysis indicated no statistically significant time × leg interaction for shear modulus in BF (F = 0.139; *p* = 0.715), SM (F = 2.554; *p* = 0.134) and ST (F = 0.338; *p* = 0.544). Similarly, there was no statistically significant time × leg interaction for PRT (F = 0.045; *p* = 0.857), active RoM (F = 0.029; *p* = 0.867) and passive RoM (F = 0.171; *p* = 0.686). The main effects of time indicated large and statistically significant increases in BF shear modulus (F = 9.583; *p* = 0.009; η^2^ = 0.42) and ST shear modulus (F = 6.725; *p* = 0.022; η^2^ = 0.34), as well as a large increase in passive RoM (F = 15.084; *p* = 0.002; η^2^ = 0.54) across both legs. Conversely, there were no time effect for SM shear modulus (F = 0.514; *p* = 0.486), PRT (F = 0.464; *p* = 0.508) and active RoM (F = 2.283; *p* = 0.155). There was also a main effect of leg for ST shear modulus (F = 7.273; *p* = 0.018; η^2^ = 0.359), as the values were consistently higher on the intervention leg.

**TABLE 1:** Descriptive statistics and analysis of variance for comparison of baseline and PostFR values.

Outcome	Leg	Baseline		Post FR		Leg			Time			Interaction		
		Mean	SD	Mean	SD	F	p	ES	F	p	ES	F	p	ES
BF	FR	9.63	2.55	10.92	2.00	0.99	0.338	0.071	9.58	0.009	0.424	0.13	0.715	0.011
	Control	9.36	2.13	10.45	2.40									
SM	FR	12.26	3.51	13.69	4.56	0.07	0.792	0.006	0.51	0.486	0.038	2.55	0.134	0.164
	Control	13.01	4.05	12.70	4.31									
ST	FR	10.32	2.69	11.60	3.84	7.27	0.018	0.359	6.72	0.022	0.341	0.38	0.544	0.029
	Control	9.48	2.59	10.43	3.54									
Passive torque	FR	10.34	7.62	10.86	7.31	0.39	0.545	0.029	0.46	0.508	0.034	0.04	0.836	0.003
	Control	9.77	5.49	10.10	6.50									
Active RoM	FR	78.61	8.94	80.54	7.83	5.25	0.039	0.288	2.28	0.155	0.149	0.02	0.867	0.002
	Control	77.11	8.06	79.18	7.48									
Passive RoM	FR	75.89	14.50	80.68	13.24	0.82	0.382	0.059	15.08	0.002	0.537	0.17	0.686	0.013
	Control	75.29	13.75	79.75	13.30									

FR, foam rolling; RoM–range of motion; SD, standard deviation; ES, effect size (partial eta-squared).

### 3.3 Effects of FR in damaged muscle state

**FIGURE 4:**
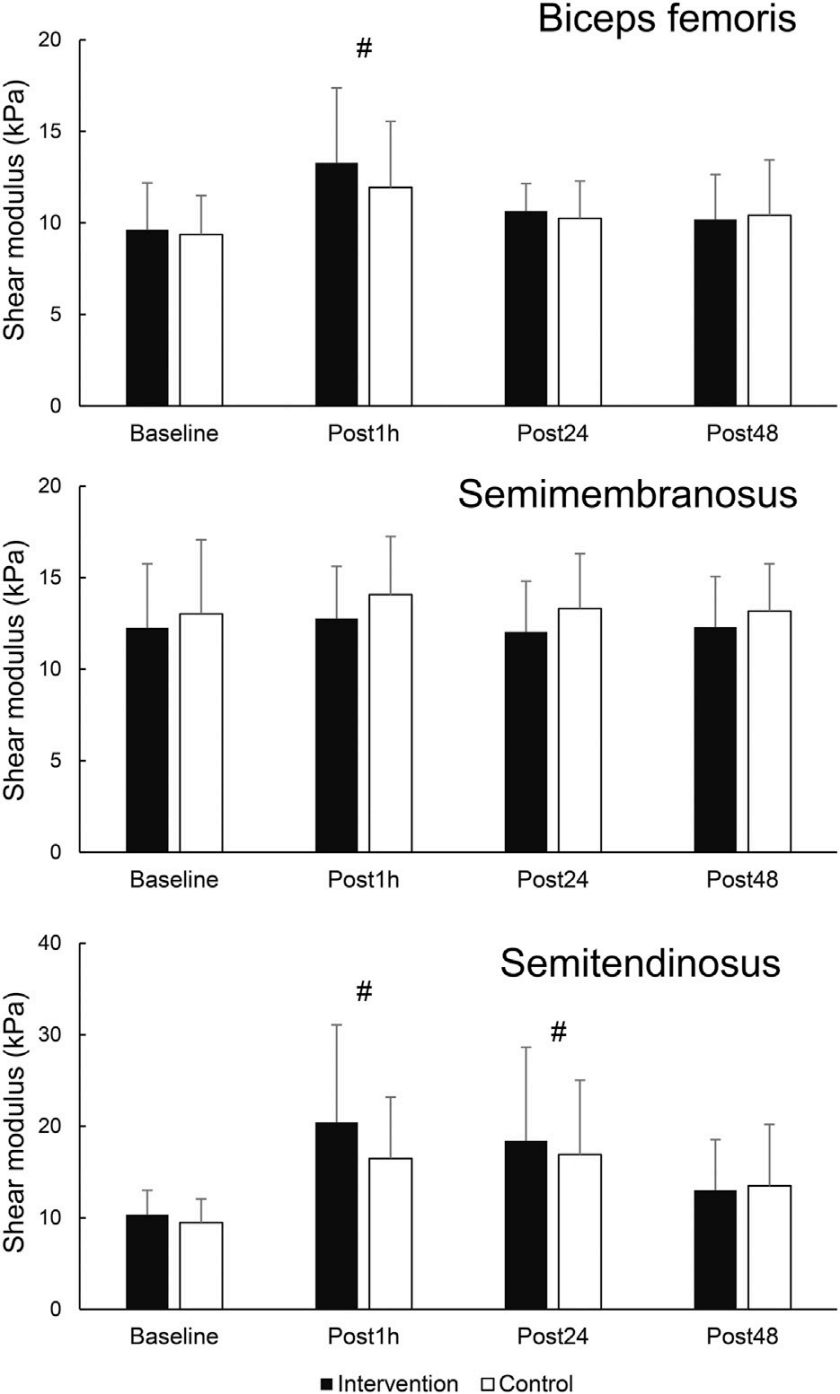
Changes in shear modulus in hamstring muscles. # - denotes statistically significant difference compared to baseline (main effect of time).

When assessing the effects of FR on damaged muscle, there were no statistically significant time × leg interactions for any of the dependent variables (F = 0.168–1.463; *p* = 0.239–0.999). Statistically significant main effect of time was present for BF shear modulus (F = 7.277; *p* = 0.001; η^2^ = 0.36). Post-hoc tests indicated an elevation of BF shear modulus at Post1h (*p* = 0.003) compared to baseline, but not at later assessments (*p* = 0.076–0.219). Similarly, there was a statistically significant main effect of time for ST shear modulus (F = 10.025; *p* < 0.001; η^2^ = 0.44), with *post hoc* tests indicating an elevation at Post1h (*p* < 0.001) and Post24 h (*p* = 0.002) compared to baseline, but not at Post48 h compared to baseline (*p* = 0.051). SM shear modulus did no exhibit any time effects (F = 0.591; *p* = 0.624). The results are displayed in Figure 4.

**FIGURE 5:**
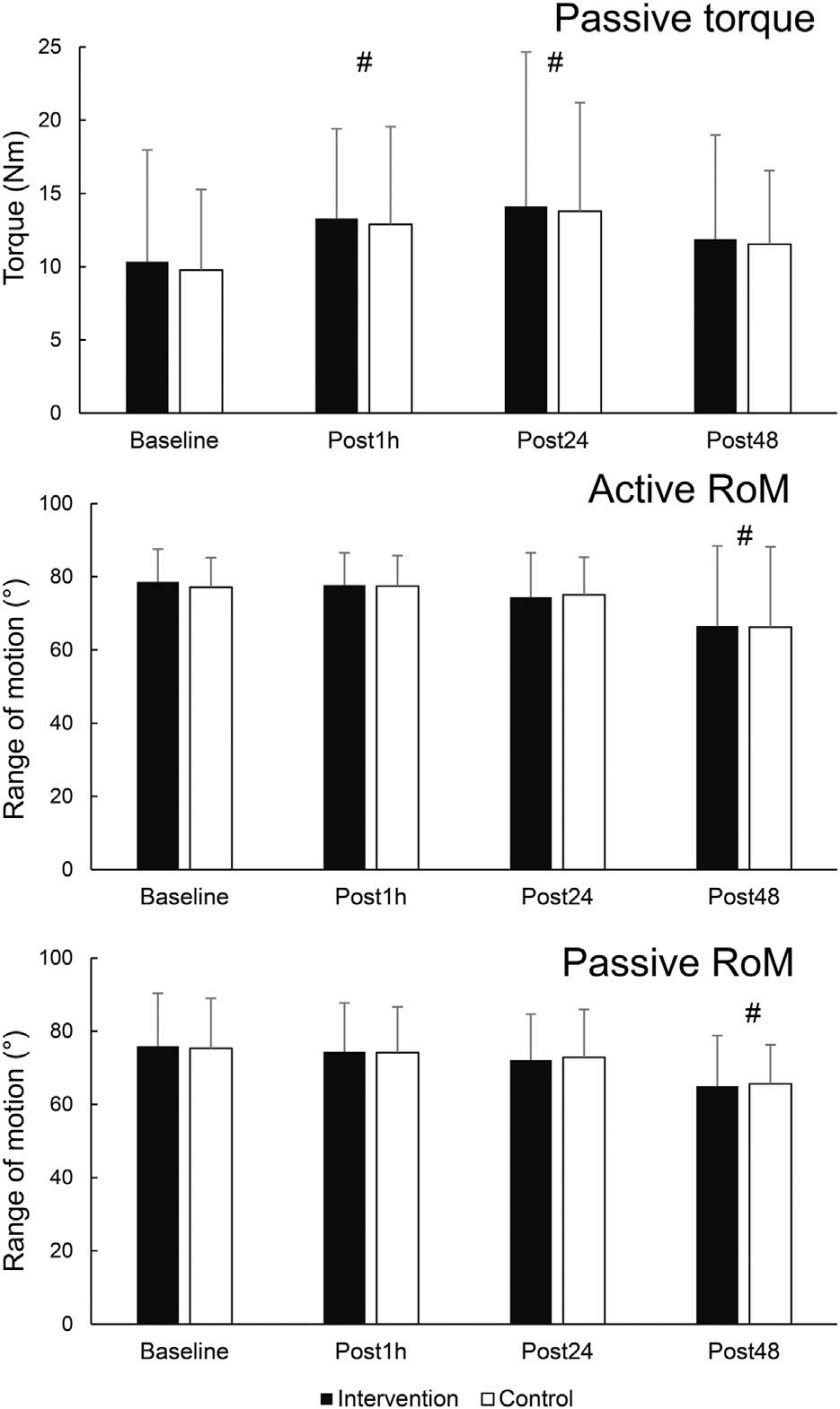
Changes in secondary outcome variables. # - denotes statistically significant difference compared to baseline (main effect of time).

The results for secondary variables are shown in Figure 5. There was a statistically significant effect of time for PRT (F = 3.978; *p* = 0.014; η^2^ = 0.23). Post-hoc tests indicated an elevation of PRT at Post1h (*p* = 0.031) and Post24 h (*p* = 0.026) compared to baseline, but not at Post48 h compared to baseline (*p* = 0.230). There was also a main effect of leg for SM shear modulus (F = 6.518; *p* = 0.024; η^2^ = 0.333), as the values were consistently higher on the control leg. Statistically significant effect of time on active RoM was also present (F = 5.872; *p* = 0.002; η^2^ = 0.31), with *post hoc* tests indicating no change at Post1h nor Post24 h compared to baseline (*p* = 0.891 and 0.198, respectively), and a statistically significant decrease at Post48 h (*p* = 0.031). Similarly, a statistically significant effect of time was present for passive RoM (F = 10.701; *p* < 0.001; η^2^ = 0.45), with *post hoc* tests indicating no change at Post1h nor Post 24 h compared to baseline (*p* = 0.367 and 0.070, respectively), and a statistically significant decrease at Post48 h (*p* = 0.001).

Finally, pain/soreness analysis indicated no difference between the legs (interaction: F = 0.650; *p* = 0.435; main effect of leg: F = 0.403; *p* = 0.537), while the results were higher during Post48 compared to Post24, as shown by statistically significant time effect (F = 16.139 *p* = 0.001; η^2^ = 0.55).

### 3.4 Correlations among changes in outcome variables

Correlation analysis indicated a possible moderate relationship between ST shear modulus changes and active RoM changes (i.e., lower stiffness being associated with larger RoM) (r = 0.55; *p* = 0.043), however, this correlation cannot be statistically confirmed while controlling for type 1 error (adjusted *p* = 0.086). There were no correlations in changes among different dependent outcome variables from baseline to Post1h (r ≤ 0.27; *p* ≤ 0.098), Post24 h (r ≤ 0.31; *p* ≤ 0.076) and Post48 (r ≤ 0.34; *p* ≤ 0.059).

## 4 Discussion

The aim of this study was to explore the impact of a shout bout of FR on hamstrings muscle stiffness, focusing on both non-damaged and post-EIMD muscle states. Our primary objective was to assess changes in shear modulus, a measure of muscle stiffness, during the recovery phase. Our results indicated that while FR did not significantly influence the shear modulus in non-damaged muscles, it also did not markedly alter muscle stiffness after the EIMD. There were also no differences between the FR and control leg regarding PRT, RoM and muscle soreness. Therefore, our alternative hypothesis was rejected. We also assessed changes in muscle stiffness through PRT and the absence of significant interactions from both measures (shear modulus and PRT) further enhances the credibility of our findings. These findings suggest a limited role of a short bout of FR in modifying muscle stiffness during recovery from EIMD, and suggest that the beneficial effects of FR reported in previous studies might be explained by central mechanisms rather than direct alterations in muscle properties. However, due to potential contralateral effects of FR, the results should be interpreted with caution.

The primary finding of this study is that FR does not affect muscle stiffness post-EIMD. In addition to the direct effects on muscle stiffness, we speculated about possible role of water migration within the muscle tissue as a response to FR. Recent literature suggests that changes in muscle properties may not solely be attributed to alterations in muscle stiffness but could also involve fluid dynamics, such as transient oedema, which may influence the shear modulus measurements post-EIMD (Proske and Morgan, 2001; Green et al., 2012; Agten et al., 2017). One plausible explanation for the lack of observed differences in muscle stiffness between FR and control legs is the potential influence of systemic mechanisms, such as pain modulation, rather than direct mechanical effects on muscle tissue. This theory aligns with research suggesting that the benefits of FR may be predominantly driven by neural mechanisms rather than alterations in muscle properties (Konrad et al., 2023). However, the exact mechanisms contributing to these effects are not yet fully elucidated. Jay et al. (2014) suggested that mechanical pressure may cause the activation of descending inhibitory pain pathways, potentially mediated through the central grey matter-opioid system and oxytocin. In addition, Drinkwater et al. (2019) found that FR improved jumping performance and reduced muscle soreness after EIMD, without changes in maximal muscle force during voluntarily and evoked contractions, which suggests pain tolerance may explain the benefits of FR for performance recovery. While the present study does not allow for definitive conclusions about the contributions of specific mechanisms, a central mechanism aligns with the acute (Wilke et al., 2020) and chronic RoM improvements after FR (Konrad et al., 2022) and reduced muscle soreness after FR in damaged muscles, as observed in several previous studies (Romero-Moraleda et al., 2019; D’Amico et al., 2020; Nakamura et al., 2021a) Therefore, the effect of FR on muscle recovery after EIMD is likely attributable to central effects, without corresponding changes in muscle stiffness or mechanical properties.

Regarding the non-damaged muscle state, there are conflicting findings in the literature concerning changes in shear modulus following FR (Morales-Artacho et al., 2017; Nakamura et al., 2021b; Reiner et al., 2021). However, the studies identifying a significant effect employed longer durations of FR (Morales-Artacho et al., 2017; Reiner et al., 2021). This suggests that a 2-min FR application might be insufficient to elicit substantial decreases in shear modulus. Previous research supports a dose-response relationship between the duration of FR and RoM improvements (Hughes and Ramer, 2019). A similar phenomenon is well-documented in static stretching, where short durations predominantly influence stretch tolerance, whereas longer durations are necessary to modify muscle stiffness both acutely and over the long term (Matsuo et al., 2013; Freitas et al., 2018). Nevertheless, a 2-min FR application was sufficient to induce local reductions in quadriceps stiffness (Schroeder et al., 2021). This finding highlights the need for further research to explore the impact of varying volumes of FR on muscle shear modulus.

The interpretation of results in the present study is complicated by the potential for contralateral effects, which presents a notable limitation of our study design. Contralateral effects, where an intervention applied to one limb can influence the other, uninvolved limb, are well-documented for FR (Konrad et al., 2023). In our study, employing a within-participant design where one leg received the FR intervention and the other served as a control, the possibility of contralateral influence could mask the effects of FR on the treated leg. However, while contralateral effects are anticipated for pain modulation, stretch tolerance, and therefore RoM (Kelly and Beardsley, 2016; Killen et al., 2019), no study to date has investigated the contralateral effects of FR on shear modulus. Studies exploring static stretching suggested that the most likely mechanism for the increased contralateral RoM is increased stretch tolerance, without changes in muscle mechanical properties (da Silva et al., 2015; Behm et al., 2019). Therefore, we can currently assume that the contralateral effects of FR might also arise from central rather than peripheral mechanisms. If this is the case, the lack of observed changes in stiffness in our study would not be due to the masking effects of contralateral influences. Nevertheless, to better isolate local and systemic influences, we recommend that future research adopts a parallel-group design.

Additional limitations of the study need to be discussed. The findings are based on a relatively small sample of 14 healthy adults. This limits the generalizability of the results to broader populations, including different age groups, athletic populations, or individuals with specific health conditions. Moreover, some outcome measures demonstrated low reliability across repetitions at specific assessment points, potentially compromising the accuracy of our findings. While the study hypothesizes about central mechanisms being responsible for the effects of FR, there was no direct investigation into the physiological or neurological mechanisms involved, which also warrants further research. Furthermore, while the use of shear modulus obtained through shear wave elastography is recognized as a valid measure of muscle stiffness, the measurement was limited to a single location in each muscle and assessed in only one participant configuration. Consequently, this approach failed to capture potential variations in stiffness across the entire muscle or at various muscle lengths. This limitation is particularly important to consider, as the responses of shear modulus to eccentric exercise are known to vary depending on muscle length and region (Lacourpaille et al., 2017; Xu et al., 2019; Goreau et al., 2022). Despite this, the similar behaviour of shear modulus to PRT supports the robustness of our findings. Furthermore, it should be noted that both the participants and the examiners were aware of the condition being tested. This lack of blinding in FR treatments is expected to continue as a fundamental challenge in conducting research in this area. Finally, this study did not account for changes in water content within the muscle tissue, which could serve as another limitation. Considering the potential impact of transient oedema on shear modulus, future research should explore the interplay between foam rolling, water migration, and muscle stiffness to provide a more comprehensive understanding of the recovery mechanisms post-EIMD.

## 5 Conclusion

This study investigated the effects of FR on muscle stiffness in both non-damaged and EIMD states. Our findings indicated no significant change in muscle stiffness as measured by shear modulus in response to FR, both in non-damaged and EIMD states. These results suggest that the benefits of FR during recovery, as reported in previous literature, may not be due to direct mechanical modifications in muscle properties. Instead, the potential role of central mechanisms, such as pain modulation, is more likely and warrants further investigation.

## Data Availability

The raw data supporting the conclusion of this article will be made available by the authors, without undue reservation.
