# Supplementary material for: Effects of foam rolling on hamstrings stiffness in damaged and non-damaged muscle states
Source: Front Physiol. 2024 Apr 8;15:1396361. doi: 10.3389/fphys.2024.1396361 (PMC11033509; doi:10.3389/fphys.2024.1396361)
Supplement: Supplementary file 1 [file Table1.DOCX]

**Supplementary file 1.** Reliability of the outcome measures.

| **Variable** | **Time** | **Repetition 1** | | **Repetition 2** | | **Repetition 3** | | **ICC** | | | **CV** | | |
| --- | --- | --- | --- | --- | --- | --- | --- | --- | --- | --- | --- | --- | --- |
|  |  | Mean | SD | Mean | SD | Mean | SD | ICC | 95%CI | | CV | 95%CI | |
| BF - Intervention Leg (kPa) | PRE | 9.39 | 2.84 | 9.86 | 2.54 | / | / | 0.83 | 0.54 | 0.94 | 12.49 | 9.05 | 20.12 |
|  | POST-1h | 12.97 | 3.34 | 13.63 | 4.87 | / | / | 0.92 | 0.77 | 0.97 | 9.75 | 7.07 | 15.71 |
|  | POST-24h | 10.68 | 1.73 | 10.62 | 1.88 | / | / | 0.40 | -0.14 | 0.76 | 13.45 | 9.75 | 21.67 |
|  | POST-48h | 10.03 | 2.27 | 10.35 | 2.71 | / | / | 0.92 | 0.77 | 0.97 | 7.55 | 5.47 | 12.16 |
| BF - Control Leg (kPa) | PRE | 9.63 | 2.27 | 9.08 | 2.16 | / | / | 0.88 | 0.66 | 0.96 | 9.02 | 6.54 | 14.53 |
|  | POST-1h | 11.59 | 3.60 | 12.29 | 3.69 | / | / | 0.96 | 0.88 | 0.99 | 6.85 | 4.96 | 11.03 |
|  | POST-24h | 9.86 | 1.97 | 10.62 | 2.37 | / | / | 0.78 | 0.45 | 0.92 | 10.58 | 7.67 | 17.05 |
|  | POST-48h | 10.22 | 2.54 | 10.61 | 3.57 | / | / | 0.93 | 0.79 | 0.98 | 8.80 | 6.38 | 14.18 |
| ST - Intervention Leg (kPa) | PRE | 10.19 | 2.78 | 10.41 | 2.73 | / | / | 0.92 | 0.78 | 0.97 | 8.05 | 5.83 | 12.96 |
|  | POST-1h | 18.32 | 8.92 | 19.80 | 9.80 | / | / | 0.97 | 0.92 | 0.99 | 9.06 | 6.57 | 14.59 |
|  | POST-24h | 18.00 | 10.62 | 17.77 | 10.01 | / | / | 0.99 | 0.96 | 1.00 | 6.97 | 5.05 | 11.23 |
|  | POST-48h | 12.81 | 6.40 | 12.76 | 4.96 | / | / | 0.94 | 0.81 | 0.98 | 12.35 | 8.96 | 19.90 |
| ST - Control Leg (kPa) | PRE | 9.53 | 2.51 | 9.46 | 2.81 | / | / | 0.92 | 0.76 | 0.97 | 8.80 | 6.38 | 14.18 |
|  | POST-1h | 17.56 | 9.34 | 18.14 | 8.54 | / | / | 0.98 | 0.95 | 0.99 | 7.15 | 5.18 | 11.51 |
|  | POST-24h | 17.06 | 8.05 | 17.86 | 8.25 | / | / | 0.97 | 0.91 | 0.99 | 8.94 | 6.48 | 14.40 |
|  | POST-48h | 13.71 | 6.27 | 13.72 | 7.06 | / | / | 0.97 | 0.90 | 0.99 | 9.66 | 7.00 | 15.57 |
| SM - Intervention Leg (kPa) | PRE | 11.76 | 3.72 | 12.75 | 3.75 | / | / | 0.79 | 0.46 | 0.93 | 14.94 | 10.83 | 24.08 |
|  | POST-1h | 13.12 | 3.32 | 12.44 | 2.68 | / | / | 0.80 | 0.49 | 0.93 | 11.21 | 8.13 | 18.06 |
|  | POST-24h | 12.20 | 3.08 | 11.88 | 2.71 | / | / | 0.83 | 0.54 | 0.94 | 10.78 | 7.82 | 17.37 |
|  | POST-48h | 12.27 | 3.09 | 12.34 | 2.65 | / | / | 0.85 | 0.60 | 0.95 | 9.65 | 7.00 | 15.55 |
| SM - Control Leg (kPa) | PRE | 13.26 | 4.36 | 12.76 | 3.86 | / | / | 0.94 | 0.84 | 0.98 | 8.19 | 5.94 | 13.19 |
|  | POST-1h | 13.78 | 3.01 | 14.36 | 3.52 | / | / | 0.90 | 0.72 | 0.97 | 8.04 | 5.83 | 12.96 |
|  | POST-24h | 13.45 | 3.12 | 13.16 | 2.98 | / | / | 0.94 | 0.83 | 0.98 | 6.01 | 4.35 | 9.68 |
|  | POST-48h | 13.02 | 2.12 | 13.32 | 3.19 | / | / | 0.85 | 0.61 | 0.95 | 8.48 | 6.14 | 13.65 |
| Passive torque - Intervention Leg (Nm) | PRE | 9.55 | 6.68 | 9.50 | 6.65 | 9.51 | 6.67 | 1.00 | 1.00 | 1.00 | 1.31 | 1.01 | 1.91 |
|  | POST-1h | 13.00 | 6.73 | 12.78 | 6.37 | 12.80 | 6.46 | 1.00 | 0.99 | 1.00 | 3.07 | 2.37 | 4.46 |
|  | POST-24h | 13.67 | 7.29 | 13.64 | 7.29 | 13.61 | 7.28 | 1.00 | 1.00 | 1.00 | 1.60 | 1.23 | 2.33 |
|  | POST-48h | 10.61 | 4.94 | 10.65 | 5.07 | 10.62 | 4.95 | 0.99 | 0.98 | 1.00 | 4.14 | 3.19 | 6.03 |
| Passive torque - Control Leg (Nm) | PRE | 10.42 | 6.54 | 10.68 | 6.62 | 10.68 | 6.63 | 1.00 | 0.99 | 1.00 | 4.11 | 3.16 | 5.97 |
|  | POST-1h | 13.36 | 6.48 | 13.33 | 6.16 | 13.21 | 6.34 | 1.00 | 0.99 | 1.00 | 2.71 | 2.09 | 3.94 |
|  | POST-24h | 14.32 | 10.65 | 14.25 | 10.62 | 14.22 | 10.58 | 1.00 | 1.00 | 1.00 | 1.11 | 0.85 | 1.61 |
|  | POST-48h | 12.74 | 7.13 | 12.84 | 7.05 | 12.76 | 6.82 | 0.99 | 0.98 | 1.00 | 4.69 | 3.61 | 6.82 |
| Passive RoM - Intervention Leg (°) | PRE | 75.50 | 14.20 | 76.29 | 14.90 | / | / | 0.99 | 0.97 | 1.00 | 2.23 | 1.62 | 3.59 |
|  | POST-1h | 74.14 | 13.76 | 74.64 | 13.08 | / | / | 0.97 | 0.92 | 0.99 | 3.16 | 2.29 | 5.09 |
|  | POST-24h | 72.21 | 12.02 | 72.00 | 13.16 | / | / | 0.99 | 0.96 | 1.00 | 2.25 | 1.63 | 3.62 |
|  | POST-48h | 64.64 | 13.70 | 65.36 | 13.95 | / | / | 0.99 | 0.96 | 1.00 | 2.68 | 1.94 | 4.32 |
| Passive RoM - Control Leg (°) | PRE | 74.93 | 13.65 | 75.64 | 14.12 | / | / | 0.97 | 0.91 | 0.99 | 3.58 | 2.60 | 5.77 |
|  | POST-1h | 73.57 | 12.48 | 74.79 | 12.68 | / | / | 0.96 | 0.89 | 0.99 | 3.55 | 2.57 | 5.72 |
|  | POST-24h | 73.07 | 13.04 | 72.71 | 13.36 | / | / | 0.96 | 0.88 | 0.99 | 4.06 | 2.94 | 6.53 |
|  | POST-48h | 65.21 | 10.33 | 66.07 | 11.11 | / | / | 0.97 | 0.91 | 0.99 | 3.07 | 2.23 | 4.95 |
| Active RoM - Intervention Leg (°) | PRE | 77.86 | 9.61 | 78.71 | 8.95 | / | / | 0.95 | 0.85 | 0.98 | 2.96 | 2.15 | 4.77 |
|  | POST-1h | 76.64 | 9.30 | 79.21 | 7.92 | / | / | 0.97 | 0.92 | 0.99 | 1.97 | 1.43 | 3.18 |
|  | POST-24h | 72.64 | 13.26 | 75.29 | 11.76 | / | / | 0.97 | 0.92 | 0.99 | 3.01 | 2.19 | 4.86 |
|  | POST-48h | 65.07 | 22.98 | 67.36 | 21.79 | / | / | 0.99 | 0.98 | 1.00 | 3.12 | 2.26 | 5.02 |
| Active RoM - Control Leg (°) | PRE | 76.64 | 8.28 | 77.57 | 7.88 | / | / | 0.99 | 0.97 | 1.00 | 1.11 | 0.80 | 1.78 |
|  | POST-1h | 76.66 | 8.82 | 78.29 | 7.78 | / | / | 0.97 | 0.90 | 0.99 | 2.14 | 1.55 | 3.44 |
|  | POST-24h | 74.43 | 10.80 | 75.79 | 10.00 | / | / | 0.96 | 0.87 | 0.99 | 3.19 | 2.31 | 5.14 |
|  | POST-48h | 64.79 | 22.61 | 67.64 | 21.31 | / | / | 0.99 | 0.97 | 1.00 | 3.35 | 2.43 | 5.39 |
